# Supplementary material for: The causal relationship between the human gut microbiota and pyogenic arthritis: a Mendelian randomization study
Source: Front Cell Infect Microbiol. 2024 Nov 26;14:1452480. doi: 10.3389/fcimb.2024.1452480 (PMC11629706; doi:10.3389/fcimb.2024.1452480)
Supplement: Supplementary file 1 [file DataSheet1.zip › mendelian test/ebi-a-GCST90027475.csv_leave_one_out.pdf]

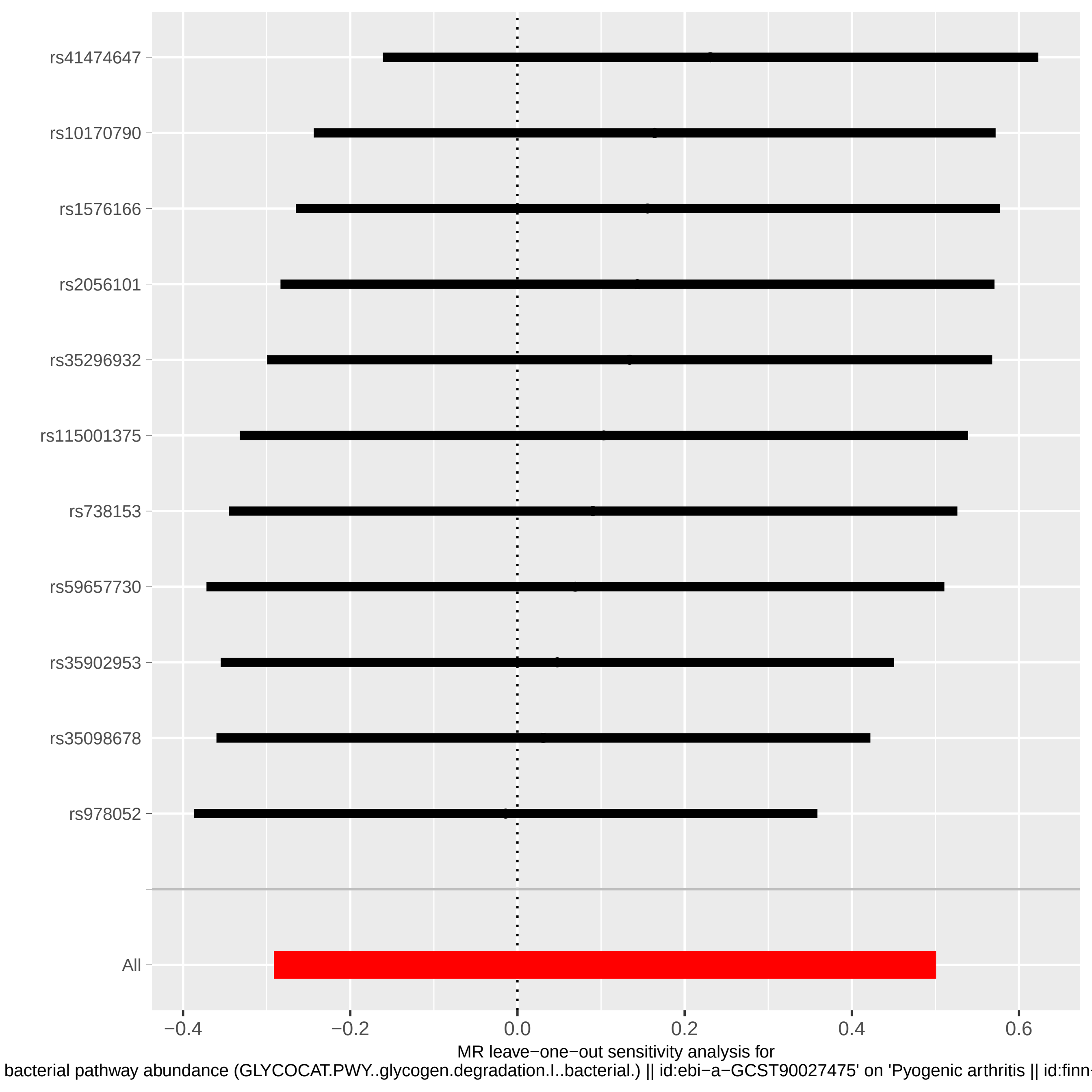

rs41474647
rs10170790
rs1576166
rs2056101
rs35296932
rs115001375
rs738153
rs59657730
rs35902953
rs35098678
rs978052
All
−0.4
−0.2
0.0
0.2
0.4
0.6
MR leave−one−out sensitivity analysis for
bacterial pathway abundance (GLYCOCAT.PWY..glycogen.degradation.I..bacterial.) || id:ebi−a−GCST90027475' on 'Pyogenic arthritis || id:finn-
